# Attempted Self-Harm with Elemental Thallium Purchased Online: Case Report with Analytical Confirmation

**DOI:** 10.1007/s13181-024-01034-9

**Published:** 2024-08-22

**Authors:** Anthony Spadaro, Andrew Sae Young Lee, Hilda Pineda, Bruce Ruck, Diane P. Calello, Howard A. Greller, Lewis S. Nelson, Mehruba A. Parris

**Affiliations:** 1 Department of Emergency Medicine, Rutgers New Jersey Medical School, Newark, NJ USA; 2 New Jersey Poison Information and Education System, Newark, NJ USA; 3 Bergen New Bridge Medical Center, Paramus, NJ USA

**Keywords:** Thallium, Heavy Metal Poisoning, Pharmacokinetics

## Abstract

**Introduction:**

Thallium is a highly toxic metal, with most publications demonstrating poisoning from thallium salts. We report on a patient with elevated serum and urine thallium concentrations from an intentional ingestion of elemental thallium purchased from the internet for self-harm.

**Case Report:**

The regional poison center was contacted about an 18-year-old man who ingested a fragment from a 100-gram bar reported to be elemental thallium. Serial serum and urine thallium concentrations were obtained. Prussian blue was started on hospital day (HD) 2. A metal fragment was seen on abdominal x-ray and removed via colonoscopy on HD3. The ingested fragment was analyzed via inductively coupled plasma mass spectrometry (ICP-MS) and found to be 87.0% elemental thallium. The initial serum thallium concentration obtained on HD1 was 423.5 mcg/L (reference range < 5.1 mcg/L), which subsequently decreased to 4.5 mcg/L, 29 days after the ingestion. An initial random urine thallium concentration obtained on HD 3 was 1850.5 mcg/g creatinine (reference range < 0.4 mcg/g creatinine). The patient remained hospitalized for 23 days and, when seen in follow-up, had not developed any signs or symptoms of thallium toxicity.

**Discussion:**

Elemental thallium ingestion is a rare toxicologic exposure, with limited published clinical and analytical experience to guide management. This case report describes a patient with ingestion of elemental thallium who developed elevated serum and urine thallium concentrations and was treated with Prussian blue. Despite having elevated serum and urine thallium concentrations consistent with previous fatal exposures, more evidence is needed to understand the differences between elemental thallium and thallium salts.

## Introduction

Thallium forms highly toxic salts that were historically used to treat tuberculosis and as a rodenticide, and more recently as an agent used in homicides [1–4]. Descriptions of the clinical course and recommendations for the management of patients exposed to elemental thallium is almost entirely based on literature from exposures to inorganic thallium salts [1]. However, little is known about the toxicity and clinical course of patients with exposure to elemental thallium, and how it may differ from those from thallium salts. This distinction is important, as experience with other metal exposures demonstrates, toxicity may vary by the physical and chemical state of the metal, as in the case of carbonyl iron which is less toxic than ionic iron [5]. In this case report we describe a rare case of ingestion of elemental thallium in a self-harm attempt.

## Case Report

**Image 1:**
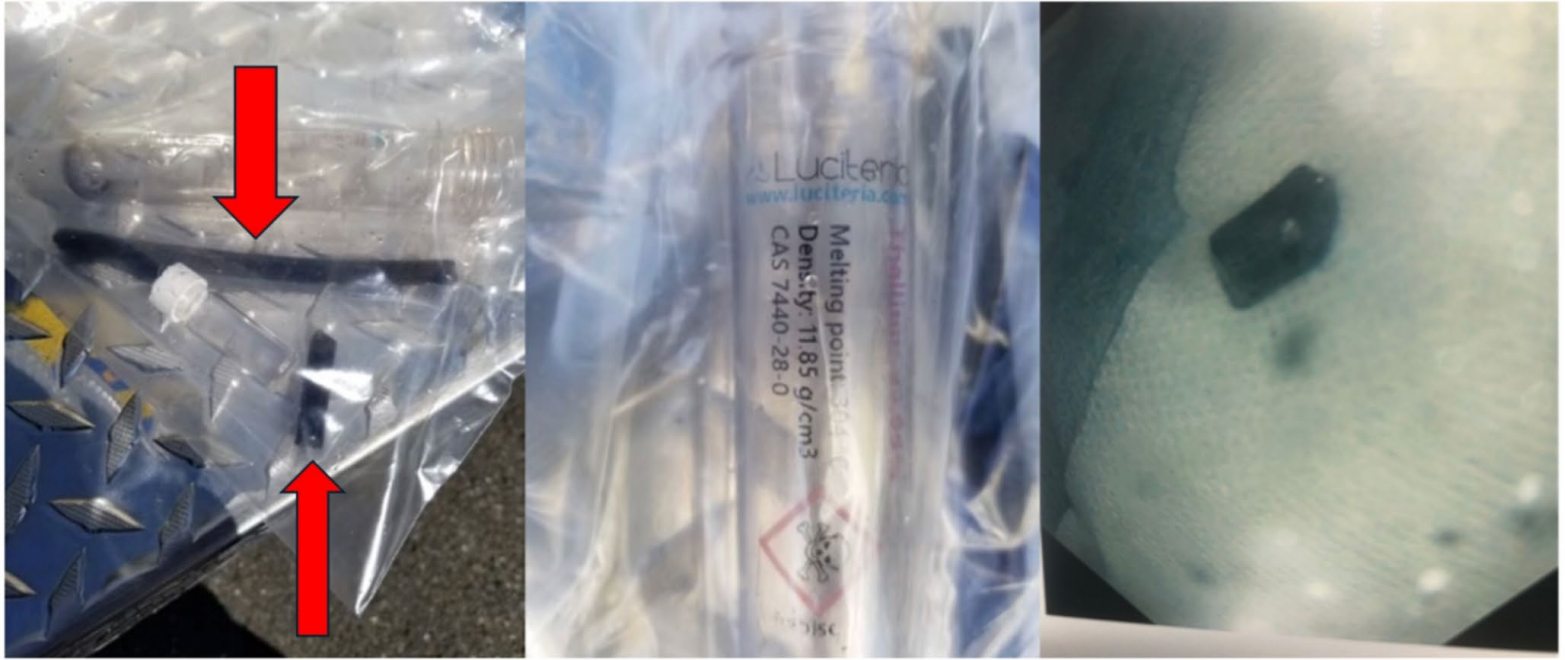
Pictures of metal fragments. **Left 1a**: Metal bar obtained from scene of ingestion (red arrows pointing to the bars added for emphasis). **Center 1b**: Vial that metal bar came in. **Right 1c**: Fragment from metal bar retrieved from colonoscopy

**Image 2:**
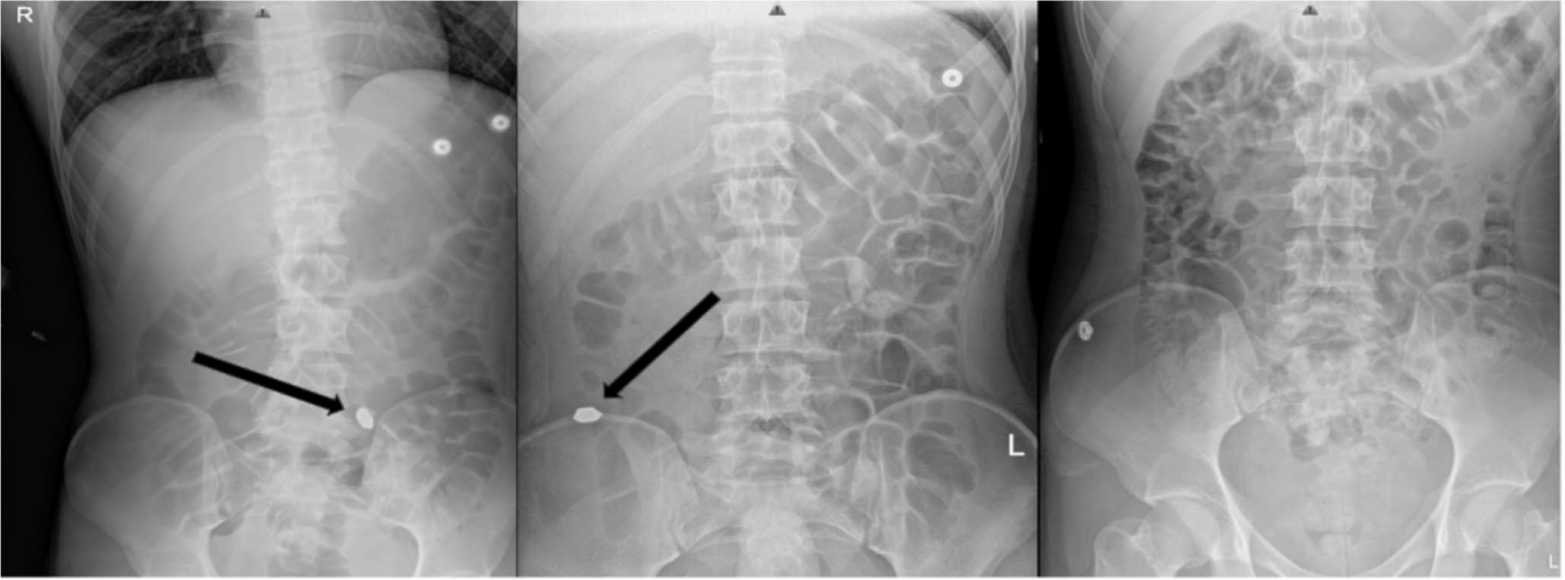
Abdominal X-rays of patient. Arrows: Radiopaque foreign body. **Left 2a**: Abdominal X-ray obtained on day of presentation, demonstrating radiopaque foreign body in left lower quadrant. **Center 2b**: Abdominal X-ray obtained on HD 2, demonstrating radiopaque foreign body in right lower quadrant. **Right 2c**: Abdominal X-ray obtained post-colonoscopy, demonstrating removal of radiopaque foreign body

An 18-year-old man was brought to the hospital from school after a self-harm attempt by ingesting a metal bar suspended in mineral oil purchased online and believed to be composed of elemental thallium (Images 1a and 1b). The patient’s initial vitals were blood pressure 128/76 mmHg, heart rate 64 beats per minute, and respiratory rate 16 breaths per minute. He had no acute complaints and a normal physical examination. Initial laboratory analysis included a normal basic metabolic panel (BMP), a normal complete blood count (CBC), and normal liver function tests (LFTs). An abdominal radiograph showed a radiopaque foreign body in the small bowel (Image 2a). On recommendation from the poison center, the patient received 30 g of activated charcoal and was admitted to the hospital for observation. In order to facilitate passage of the radiopaque foreign body, the patient was given 17 g of oral polyethylene glycol twice a day. Oral insoluble Prussian blue (Radiograms™, McGuff Pharmaceuticals), 3 g three times daily was started empirically on hospital day (HD) 2, prior to the result of any diagnostic test for thallium. Serial abdominal radiographs were performed to assess the passage of the radiopaque foreign body (Image 2b). Colonoscopy was performed on HD 3 to remove the foreign body due to non-progression on serial x-rays (Image 2c). Testing via ICP-MS by the Environmental and Chemical Laboratory Services Lab in Trenton, NJ, found that the mineral oil surrounding the metal bar contained trace amounts of thallium, 0.00856% by weight. The metal bars recovered from the scene and the patient (image 1c) appeared to be dark after being exposed to air for several days and potentially oxidized, however both were sent for testing. The metal bar obtained from the scene was tested, and determined to be 90.5% thallium by weight, and the metal bar removed from the patient was 87.0% thallium by weight. The lab was unable to further determine if the thallium present was in an elemental or ionic form. Neither sample was 100% thallium and it is unknown if the remainder of the sample was composed of other metals or reflective of weight of oxygen in thallium oxides.

.

**Fig. 1:**
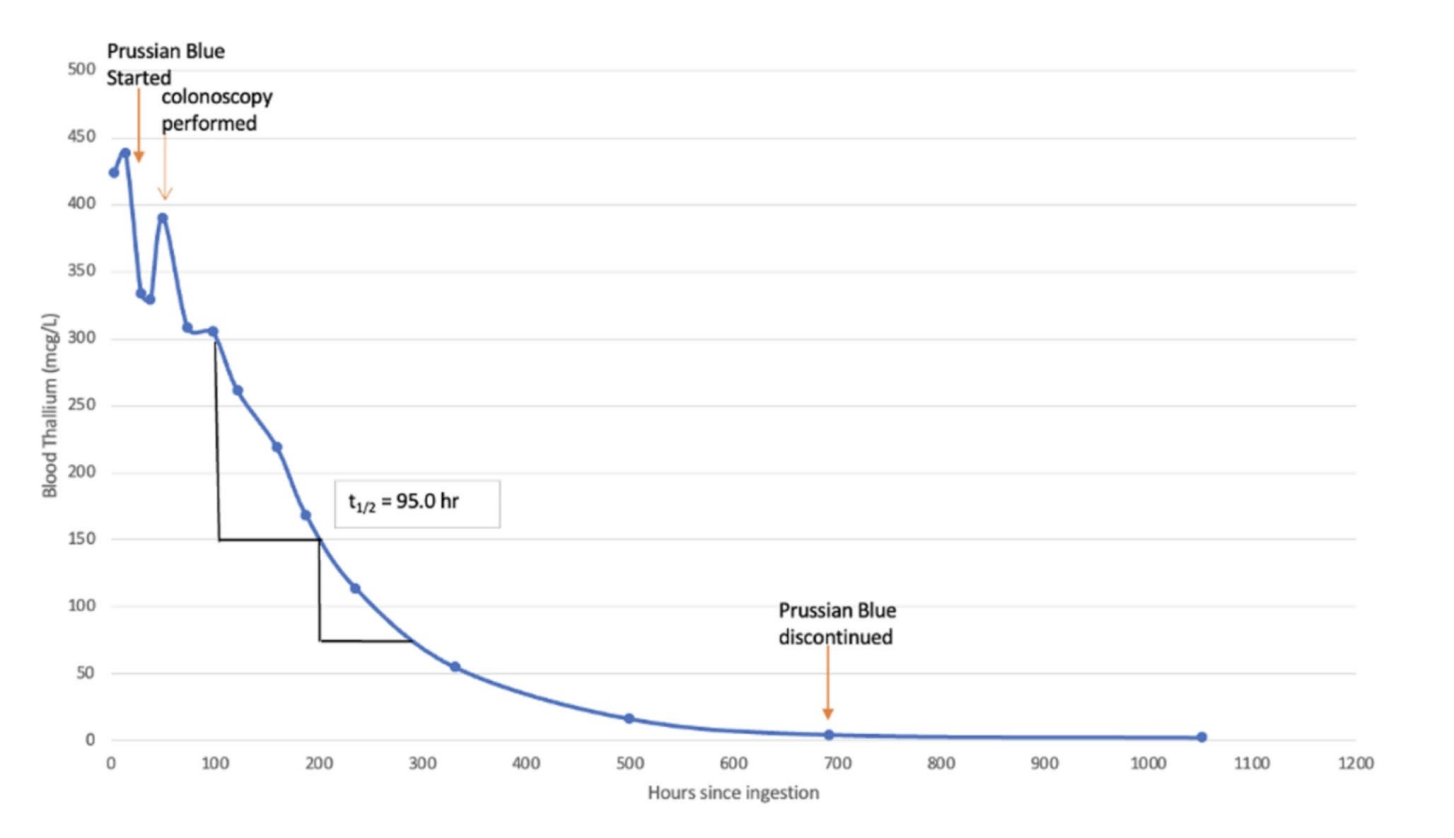
Blood thallium concentrations over time

Due to the concerning nature of this patient’s ingestion, he was kept in the hospital to observe for both the development of symptoms, and serial laboratory testing. Over his 22-day hospitalization, serum thallium concentrations declined (Fig. 1). On HD 8, the first resulted serum thallium concentration obtained three hours after ingestion was 423.5 mcg/L (reference range < 5.1 mcg/L). Random spot urine thallium concentrations were also obtained and were elevated, with an initial concentration of 1850.5 mcg thallium/g creatinine on HD 3 (reference range < 0.4 mcg/g). A 24-hour urine thallium concentration was not obtained due to difficulty collecting the sample. The patient did not develop any signs or symptoms of thallium toxicity such as alopecia, eczematous skin rashes, diarrhea, constipation, painful ascending neuropathy, ataxia, pleuritic chest pain, hypertension, tachycardia, acute kidney injury, multiorgan failure, or coma. The patient continued Prussian blue for a total of thirty days. When evaluated in medical toxicology clinic, 38 days after ingestion, the patient remained asymptomatic. After discussing with the patient and his family, it was determined that there was no ongoing source of thallium exposure in the house or occupational setting. The patient’s last detectable blood thallium concentration was 2.4 mcg/L, 44 days after ingestion. At the last outpatient evaluation, 86 days after ingestion, he remained asymptomatic, had an undetectable blood thallium concentration, and normal CBC, BMP, and LFTs. Scalp hair obtained from the patient at this visit did not reveal any obvious dark pigmentation when evaluated under a light microscope when compared to a control hair (Tuword Microscopes, 40X magnification). Consent for publication of this case was obtained and provided to the journal in accordance with JMT policy.

## Discussion

This case describes a patient who ingested elemental thallium and remained asymptomatic despite having prolonged and markedly elevated blood concentrations. The diagnosis and treatment of thallotoxicosis may be delayed because of the delayed nature of symptom development, in addition to exposures being due to unrecognized malicious intent [1–4, 6]. Interpretation of diagnostics tests are challenging since testing for thallium may be performed and result many days after exposure. In one large case series of patients with thallotoxicosis, the median time from symptom onset to admission was 13 days, and patients had symptoms along with an initial mean blood thallium concentration of 293 ng/ml [7]. In another case series, 10 patients from two families ate cakes contaminated with thallium and developed symptoms within hours [2]. These patients had blood thallium concentrations on admission that ranged from 53 mcg/L to 1700 mcg/L, although they did not present until 5–7 days after the suspected exposure [2]. From this series, 5 cases required ICU admission, 3 cases required mechanical ventilation, and 2 cases had evidence of cerebral edema on CT scan. Three of these 10 patients went on to have multi-organ failure and die. In another case series, four patients were exposed to thallium contaminated candies, and were started on therapies three days after exposure. It was estimated that these patients may have consumed up to 1 gram of thallium. While serum thallium concentrations were not reported, the patient with the most severe symptoms had a spot urine thallium concentration of 10,837 mcg/L on day 3, compared to our patient who had a spot urine thallium of 1850.5 mcg/g of creatinine on day 3. Although the patients had positive outcomes, they all developed symptoms, all four had alopecia and two had painful neuropathy [4].

Thallium is a multiorgan toxin that can cause both acute and delayed onset toxicity. Immediate early symptoms of toxicity include primarily gastrointestinal symptoms such as nausea, vomiting, diarrhea, and constipation [1, 2]. In the days to weeks after exposure, neurologic manifestation such as painful neuropathy, delirium, and coma may develop [1, 2]. Multiorgan failure with acute kidney injury, sinus tachycardia, pericardial effusion, and respiratory depression may also develop in this time frame [1]. Dermatologic manifestations such as alopecia or leukonychia striata (Mee’s lines) may develop at any time and last for a prolonged period [1, 6]. For patients who survive an acute episode of thallotoxicosis, dermatologic and neurologic manifestations may persist for weeks [1]. Thallotoxicosis is in the differential diagnosis of an unexplained peripheral neuropathy, rapid-onset alopecia, and severe multiorgan failure [7]. In this case, the patient was assessed for these signs and symptoms development while in the hospital and during outpatient follow-up. With a peak serum thallium concentration of 438.1 mcg/L, this patient had a concentration in a range similar to other patients that developed symptoms ranging from alopecia and neuropathy, to multiorgan failure and death. Direct comparison of serum thallium concentrations is challenging as there commonly is a delay in diagnosis, and the true peak serum concentration is often unknown. Additionally, asymptomatic thallium exposures are likely under-reported and corollary serum thallium concentration known to produce symptoms is not known. There may be several reasons why our patient remained asymptomatic. The patient’s exposure was known immediately, and they promptly received activated charcoal, known to adsorb thallium. They were also started on Prussian blue to enhance elimination of thallium as soon it was available to the hospital. Although this hospital was able to obtain Prussian blue rapidly, it is an uncommon antidote and there may be delays in obtaining it. Prussian blue is an oral cation exchange resin that decreases luminal thallium absorption and interferes with enterohepatic recirculation [1]. Soluble Prussian blue may adsorb thallium better than insoluble Prussian blue [8]. Prussian blue reduces the half-life of thallium and may decrease thallium content in end organs [1]. The patient also had a relatively short time to colonoscopy to remove the source thallium metal. Despite these interventions the patient still had a relatively high serum thallium concentration, and timely treatment does not entirely explain his lack of symptoms.

One unique aspect of this case was that the patient ingested what was reported to be elemental thallium, while most clinical experience is with thallium salts [1–4, 6, 7]. The thallium which this patient ingested was found to be greater than 87% thallium by weight. While the mechanisms underlying thallium toxicity are not completely known, there are several proposed explanations. Thallium ions have a similar ionic radius to potassium ions and tend to accumulate in areas of high potassium concentrations such as the nervous system, kidneys, heart, and muscle. Thallium also impairs potassium-dependent enzymes, such as pyruvate kinase, succinate dehydrogenase, and sodium potassium adenosine triphosphatase which may lead to oxidative stress and impair mitochondrial function [1, 9]. These mechanisms may explain the multisystem manifestations of thallotoxicosis as it causes widespread impairment of cellular energy utilization. Thallium has high affinity for charged sulfhydryl groups present in keratin which may explain the dermatological manifestations of thallotoxicosis [6]. As elemental thallium is not charged, it has a different radius and electrochemical interactions than thallium ions [9, 10]. It is unknown how elemental thallium may interact with the enzymes purported to be involved in the mechanisms of thallium salt toxicity.

Thallium salts have a bioavailability exceeding 90% [7]. Elemental thallium is not soluble in water [11]. While elemental thallium has absorption in the gastrointestinal tract as evidenced by this patient’s detectable blood thallium concentrations, the bioavailability of elemental thallium is unknown. The exact impact of gastrointestinal contents on elemental thallium is also not known. However, elemental thallium is known to be a highly reactive metal that oxidizes to TI_2_O or TI_2_O_3_ when exposed to air [10, 12]. The outer surface of part of the elemental thallium bar recovered from the scene appeared to have oxidized and had a black appearance rather than the silvery appearance of elemental thallium (Fig. 1a). Thallium exists in the environment as ionic thallium in either a Tl^+ 1^ or Tl^+ 3^ form [9]. pH affects the solubility of thallium oxides [13]. It is unknown how much of the thallium bar was oxidized at the time of ingestion and how this may have effected the absorption of thallium. In this patient, thallium had an estimated half-life of 95 h, compared to 40 h published elsewhere [1]. The lab performing the blood and urine thallium testing could not determine if they were detecting elemental or ionic thallium. Thus, it is unknown if this patient’s detectable thallium concentrations were elemental or ionic thallium. The thallium bar recovered from the scene and from the patient was found to be 90.5% and 87.0% thallium by weight respectively, and it is unknown if the remainder of the sample was composed of other metals or reflective of weight of oxygen in thallium oxides. This would raise the possibility that some of the thallium the patient absorbed was oxidized.

This case poses several clinical questions. Activated charcoal was initially given and then colonoscopy was performed due to failure of the thallium to progress through the gastrointestinal tract. Whole bowel irrigation may have obviated the need for colonoscopy. Gastric lavage may also be considered in patients who present shortly after ingestion, if it is suspected that there is thallium in the stomach, but was not performed here as the thallium was already past the pylorus of the stomach on the first x-ray. Due to its similar ionic radius, it has been reported that potassium supplementation may enhance thallium elimination, but this practice is not universally recommended and was not pursued in this patient [1]. Extracorporeal removal of thallium with hemodialysis or hemoperfusion may be considered in severely poisoned patients, but as this patient remained clinically stable and asymptomatic this was not pursued [1]. N-acetylcysteine in thallium toxicity has also been described but was not utilized in this case [4]. The use of serum thallium concentrations to guide management is not well described. In this case serum thallium concentrations took several days to result, limiting the real-time utility in guiding management of the patient. Case reports describe administering 150–250 mg/kg/day of Prussian blue divided into 2–4 doses, while the prescribing information recommends 3 g every 8 h [1, 2, 6]. The end points for Prussian blue therapy are not well described, with some authors advocating for treatment with Prussian blue until a 24-hour urine thallium concentration falls below 0.5 mg/day [1]. In this case, Prussian blue was continued for 30 days as recommended by the manufacturer [14]. Prussian blue is known to enhance elimination of thallium and it was decided to continue it until the patient’s blood thallium concentrations approached undetectable levels [15]. It is not known if shorter regimens could be used for patients without symptoms.

Although this patient never developed any evidence of thallium toxicity, it would be premature to conclude that elemental thallium is not toxic. The lethal dose of thallium salts has been reported to be 10–15 mg/kg, and even a small ingestion can be lethal [2]. All patients with suspected or confirmed thallium toxicity should be treated aggressively. Until there is more evidence describing elemental thallium ingestion, we recommend that it should be treated similarly to thallium salt ingestion. Given the rarity of elemental thallium ingestions, animal models would be necessary to further explore how elemental thallium ingestion may be different from thallium salt ingestion.

While suicidal ingestion of thallium remains rare, the ease of obtaining the source product online is of additional concern. The website from which the thallium was purchased appeared to sell various elements for individuals who are interested in collecting. The commercial use of thallium salts in rodenticides was banned in the United States in 1975, however online retail of thallium is not well regulated, and clinicians should be aware that patients can access toxic metals from online retailers.

## Conclusion

This case describes a patient that ingested elemental thallium in a self-harm attempt. Despite having detectable blood and urine thallium concentrations for over a month, this patient did not develop any evidence of thallium toxicity. While early and aggressive therapy may have contributed to this positive outcome, more evidence is needed to understand how the toxicity of elemental thallium differs from thallium salts.

## Declarations

### Prior presentations

Data in this manuscript were previously presented at ACMT’s Annual Scientific Meeting, Washington, DC, 2024.
